# Online Gain Adaptation of Whole-Body Control for Legged Robots with Unknown Disturbances

**DOI:** 10.3389/frobt.2021.788902

**Published:** 2022-01-07

**Authors:** Jaemin Lee, Junhyeok Ahn, Donghyun Kim, Seung Hyeon Bang, Luis Sentis

**Affiliations:** ^1^ Department of Mechanical Engineering, University of Texas at Austin, Austin, TX, United States; ^2^ College of Information and Computer Sciences, The University of Massachusetts Amherst, Amherst, MA, United States; ^3^ Department of Aerospace Engineering and Engineering Mechanics, University of Texas at Austin, Austin, TX, United States

**Keywords:** whole-body control, gain adaptation, external disturbances, legged robot control, stability analaysis

## Abstract

This paper proposes an online gain adaptation approach to enhance the robustness of whole-body control (WBC) framework for legged robots under unknown external force disturbances. Without properly accounting for external forces, the closed-loop control system incorporating WBC may become unstable, and therefore the desired task goals may not be achievable. To study the effects of external disturbances, we analyze the behavior of our current WBC framework *via* the use of both full-body and centroidal dynamics. In turn, we propose a way to adapt feedback gains for stabilizing the controlled system automatically. Based on model approximations and stability theory, we propose three conditions to ensure that the adjusted gains are suitable for stabilizing a robot under WBC. The proposed approach has four contributions. We make it possible to estimate the unknown disturbances without force/torque sensors. We then compute adaptive gains based on theoretic stability analysis incorporating the unknown forces at the joint actuation level. We demonstrate that the proposed method reduces task tracking errors under the effect of external forces on the robot. In addition, the proposed method is easy-to-use without further modifications of the controllers and task specifications. The resulting gain adaptation process is able to run in real-time. Finally, we verify the effectiveness of our method both in simulations and experiments using the bipedal robot *Draco2* and the humanoid robot *Valkyrie*.

## 1 Introduction

Stability analysis for efficient whole-body control (WBC) of humanoid robots is important to execute robustly multiple tasks in bipedal and humanoid robots. Most WBC approaches face difficulty ensuring the stability at the closed loop systems due to intricate control structures. For this reason, bipedal and humanoid robot stability is frequently studies in task-space, based on constant feedback gains. However, these feedback gains defined a priori might be inappropriate to track the planned motions robustly and stabilize the robot’s behaviors under unknown external disturbances. This paper proposes an online gain adaptation method based on stability analysis of the closed-loop robotic system *via* WBC. A basic assumption of our problem is that there are no force/torque sensors to measure both contact forces and external force disturbances. Our online gain adaptation approach utilizes three methods: *1*) a WBC controller, dubbed Whole-Body Locomotion Controller (WBLC), *2*) a Centroidal dynamic model, and *3*) various approximation techniques.

### 1.1 Whole-Body Control

Legged humanoid robots need to deliver stable and robust legged manipulation (also referred to as loco-manipulation) behaviors while operating in unstructured environments. For this purpose, Whole-Body Control (WBC) enables the generation of control commands for tracking multiple task trajectories effectively Khatib et al. (2004). Most WBC frameworks rely on dynamic models of the robot and classical control laws such as proportional-derivative (PD) control Feng et al. (2015) or impedance control Albu-Schäffer et al. (2007). Based on these requirements, projection-based WBC approaches have been widely used due to their intuitive, concise, and computationally efficient form. However, more versatile algorithms are frequently needed to consider inequality and unilateral constraints Lee et al. (2012) such as Contact Wrench Cone (CWC) constraints Caron et al. (2015). Optimization-based approaches have been widely used to incorporate inequality constraints explicitly Escande et al. (2014); Hong et al. (2020); Feng et al. (2014); Wiedebach et al. (2016); Kim et al. (2020). However, there is a major challenge for these optimization-based WBC approaches: how can we ensure the desired task hierarchy and how can we guarantee system stability when there exist uncertain external forces?

A well-known method to enforce task priorities employs hierarchical quadratic programming (HQP) Escande et al. (2014); Hong et al. (2020). In Feng et al. (2014); Wiedebach et al. (2016), the authors employ a single quadratic program (QP) with different weighted cost terms to impose a task hierarchy. These types of controllers that employ a single QP process reduce the number of optimizations compared with HQP and enforce the task hierarchy in an implicit manner; however, the weights are heuristic, resulting in occasional violations of the priorities. Often, there is a discrepancy between the desired configuration and the result from the WBC optimization. The joint acceleration, which is one of the results from the WBC optimization process, can be numerically integrated to resolve this problem Kuindersma et al. (2016); Koolen et al. (2016); Ahn et al. (2021). Alternatively, this problem can be solved by directly re-optimizing the trajectories considering dynamic reachability Lee et al. (2020). Another approach is to have WBC incorporate both internal and external force/torque measurements *via* embedded sensors within the mechanical actuation/transmission system Nori et al. (2015). These sensors can be used to improve the stability of the robots *via* their WBC controllers.

**FIGURE 1:**
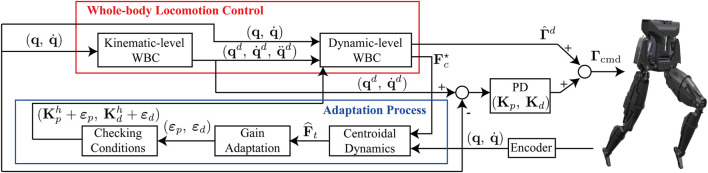
Block diagram of the proposed approach: The proposed approach includes various feedback loops, inside and outside the whole-body locomotion controller (WBLC) Kim et al. (2020). Our adaptive approach is tasked with modifying feedback gains inside WBLC.

In general, it is complex to analyze the stability of robots controlled by the WBC approaches. For instance, stability of a priority-based kinematic control approach is verified based on Lyapunov stability in Antonelli (2009). Also, it is shown that the robot controlled by the operational space control framework is asymptotically stable Dietrich et al. (2018). However, the above stability analysis studies do not consider floating-base dynamics of the legged robot and the presence of unknown external disturbances. Compared with the above stability analysis which are applied to projection-based approaches, the stability analysis of optimization-based WBC approaches is more complicated to perform when considering external perturbations. We previously proposed an optimization-based WBC method dubbed WBLC, that combines both a projection-based controller and an optimization technique to enforce task priorities while satisfying inequality constraints Kim et al. (2020). The WBLC framework combines a whole-body kinematic controller and a whole-body dynamic controller to compute joint position, velocity, acceleration, and torque references in real-time as shown in Figure 1. WBLC has successfully demonstrated dynamic locomotion of passive ankled robots. However, the robustness of WBLC regarding the external disturbances mostly relies on the experts’ parameter tuning in experiments. Also, we did not address the theoretic stability analysis in our previous work Kim et al. (2020). In this paper, we will analyze here our WBLC framework for ensuring stability and tracking performance under external forces. This is key since if WBLC is affected by unknown external forces, the robot could easily lose balance.

### 1.2 Robots With External Disturbances

Compared to other methods which focus on push recovery, here we focus on adaptive control as a means to deal with external forces. Some push recovery methods employ linear MPC to obtain CoM and foot trajectories based on the Zero Moment Point (ZMP) dynamics to re-plan walking behaviors for disturbance recovery Mason et al. (2018). The desired recovery force associated with the ZMP condition is converted to CoM positions Wang et al. (2014). It has been widely investigated how to determine stepping motions for balance recovery such as Push Recovery Model Predictive Control (PR-MPC) Stephens and Atkeson (2010b) and Capture Point control Pratt et al. (2006). Although these strategies have accomplished successful results, they are based on motion generation instead of modifying feedback gains or impedances. However, the latter strategy considered in this paper can provide a layer of additional robustness. Indeed, it is not always needed to perform an additional stepping motion to recover from external forces. If the external or contact forces are not adequately compensated for or there exist unknown external forces, the controllers may be unstable regardless of the recovery strategy Nakanishi et al. (2008).

One solution to this problem is to compensate for external forces. This has been often studied as a compliant control problem for balancing despite the external forces Hyon et al. (2007); Stephens and Atkeson (2010a); Ott et al. (2011); Herzog et al. (2016). A key idea of the above researches is to obtain proper ground reaction forces keeping the balance against the external force then the forces are utilized to compute the control command torque Hyon et al. (2007); Ott et al. (2011). LQR is employed for better momentum control of a torque-controlled humanoid and they show robust performance on balancing in the face of unknown disturbances Herzog et al. (2016). A common point of the above researches is that they rely on constant gains or impedances. Although the above researches have accomplished practical balancing ability, the stability of the controlled robots are not proven at the actuation level yet when the external forces are applied to the robots. Furthermore, they also need to modify or replan the task specifications such CoM and angular momentum for keeping the robot’s balance. By contrast, our work does not require this replanning step.

Another solution to the above problem is to directly measure external forces/moments using an F/T sensor Käslin et al. (2018) or internal, tactile, and F/T sensors, simultaneously Ivaldi et al. (2011). However, it is normally challenging to attach F/T sensors everywhere in the robot’s body. An alternative method is to accurately estimate external forces and the corresponding ground reaction forces relying on the dynamic model of the robot Xin et al. (2018). For humanoid robotic applications, an external force/moment can be estimated based on an extended Kalman filter using whole-body dynamics, force sensor on the feet, and an IMU Benallegue et al. (2018). In some cases, external forces can be measured using force-sensing resistors Hawley et al. (2019) on the feet. We can also deploy adaptive control approaches for stabilizing the robots without direct estimation of the external forces. A robust and adaptive control approach was proposed without using the robot’s dynamic model to guarantee stable control performance under uncertain disturbances Lee et al. (2016). This approach shows the robustness of WBC with respect to impact disturbance at the task level.

### 1.3 Contributions

Overall, this paper proposes a unique gain adaptation approach for WBLC applied to legged robots under both contact and external forces. More specifically, we aim to enhance the robot’s balance and robust performance by guaranteeing feedback system stability under unknown external forces. To do that, we leverage the WBLC as proposed in Kim et al. (2020). Then, we approximately decouple the joint-space dynamics and analyze their closed-loop stability. Compared to other approaches such as Lee et al. (2016), we adapt the feedback gains to make the closed-loop system critically damped at the joint level. This results in four contributions.

• Firstly, the proposed adaptation approach is very intuitive and straightforward to be implemented. Our method does not require any modification of the task specifications defined a priori. Without any significant modifications to the controller and planners, it is also possible to employ classical WBC methods Kim et al. (2020) for controlling the robot in the presence of unknown external forces.
• Second, the proposed WBLC approach also improves task tracking performance with respect to external disturbances. This improvement results in the robot’s ability to improve its balance and provide more robust bipedal walking.
• Third, we provide a joint-level control stability study of robots perturbed by external forces. There exist a number of studies considering the stability of legged robots performed at the force or task/operational space level. These types of stability analysis cannot ensure whole-body stability involving actuation effects since those take place at the joint level.
• Lastly, the proposed online gain adaptation method is designed to be easy-to-use. We do not need to rely on F/T sensors and a complicated estimation process to stabilize the robot system due to our use of centroidal robot dynamics.

In this paper, our primary task is to control the position of the CoM, keeping balance in the double support phase and to stably walk against the external disturbances. Many studies on legged robots such as push recovery and reactive planning have addressed keeping balance while standing and walking. Regarding reactive planning processes, they do not change low-level controller feedback gains as we do and instead modify the desired task specifications or trajectories in the presence of the perturbations. Therefore, in such methods, it is hard to guarantee the closed-loop stability of the robot while tracking the desired task trajectories. By contrast, our method stabilizes the robot against the external disturbance by adapting the feedback gains without further modification of task trajectories. For this reason, we can ensure the closed-loop stability of the robot while the external disturbance is applied to the robot. Our proposed approach is therefore simpler and more intuitive than reactive planning approaches and it can also be used in combination with them to enhance their performance.

The remainder of this paper is organized as follows. Section 2 explains the preliminaries related to a QP-based WBC approach, e.g., WBLC and the Centroidal Dynamic model. The proposed approach is explained in Section 3. In Section 4, we apply the proposed adaptive approach to a legged robot *Draco2* to show the effectiveness of the proposed method in numerical simulations and hardware experiments.

## 2 Preliminaries

In section 2.1, we first review our previous WBLC. Then, in section 2.2, we thoroughly analyze the closed-loop behavior of robots with no external perturbations aside from the ground forces needed to balance.

### 2.1 QP-Based Whole-Body Locomotion Control

We review here the structure of our previous WBC controller. Given a configuration space $Q\subseteq R^{n}$ and an input space $U\subseteq R^{n_{u}}$ , the rigid-body dynamics of a robot is described as follows:

$$M\left(q\right)\ddot{q}+B\left(q,\dot{q}\right)=S^{⊤}\Gamma +J_{c}^{⊤}\left(q\right)F_{c}$$ (1)

Where q∈Q , $M\left(q\right)\in S_{++}^{n}$ , $B\left(q,\dot{q}\right)\in R^{n}$ , and Γ∈U denote the joint position vector, mass/inertia matrix, sum of Coriolis/Centrifugal and gravity forces, and torque command, respectively. Considering six DOFs virtual joints for the floating base, *n*_*u*_ = *n* − 6, we can define the selection matrix $S=\left[\begin{array}{cc} 0_{n_{u}\times 6} & I_{n_{u}\times n_{u}} \end{array}\right]\in R^{n_{u}\times n}$ . $F_{c}\in R^{6m}$ is a vertically concatenated contact force vector and the corresponding contact Jacobian is represented as $J_{c}\left(q\right)\in R^{6m\times n}$ where *m* is the number of contacts. A standard feedback control scheme can be designed in the joint space using a PD control law:

$$\Gamma_{\text{cmd}}=SM\left(q\right)\left(K_{p}e_{q}+K_{d}{\dot{e}}_{q}\right)+{\hat{\Gamma }}^{d}$$ (2)

Where **e**_**q**_ = **q** ^*d*^ − **q**, and ${\dot{e}}_{q}={\dot{q}}^{d}-\dot{q}$ . Also, $q^{d}\in Q$ , ${\dot{q}}^{d}\in R^{n}$ , $K_{p}=\mathrm{diag}\left(k_{p_{1}},\ldots ,k_{p_{n}}\right)$ and $K_{d}=\mathrm{diag}\left(k_{d_{1}},\ldots ,k_{d_{n}}\right)$ denote desired joint position, desired joint velocity, proportional gain matrix and derivative gain matrix, respectively. ${\hat{\Gamma }}^{d}$ represents a feedforward torque command generated by the *Dynamic-level WBC* control block shown in Figure 1. Using a hierarchical inverse kinematic control scheme, we can compute **q** ^*d*^ and ${\dot{q}}^{d}$ given task specifications, **x** ^*d*^, ${\dot{x}}^{d}$ , and ${\ddot{x}}^{d}$ . Let us consider *N* hierarchical tasks in a lexicographic order. The desired joint position is obtained by **q** ^*d*^ = **q** + Δ**q** where $\Delta q=\sum_{k=1}^{N}\Delta q_{k}$ and

$$\begin{array}{cc} \Delta q_{k} & =J_{k|k-1}{\left(q\right)}^{+}\left(e_{x_{k}}-J_{k}\left(q\right)\Delta q_{k-1}\right),\\ N_{k}\left(q\right) & =N_{k-1}\left(q\right)-J_{k|k-1}{\left(q\right)}^{+}J_{k|k-1}\left(q\right) \end{array}$$ (3)

Where **J**_*k*|*k*−1_(**q**) = **J**_*k*_(**q**)**N**_*k*−1_(**q**), $e_{x_{k}}=x_{k}^{d}-x_{k}$ and **N**_0_(**q**) = **I**. In addition, ${\dot{q}}^{d}$ and ${\ddot{q}}^{d}$ can be simply computed as ${\dot{q}}^{d}=\sum_{k=1}^{N}{\dot{q}}_{k}^{d}$ and ${\ddot{q}}^{d}=\sum_{k=1}^{N}{\ddot{q}}_{k}^{d}$ where

$$\begin{array}{cc} {\dot{q}}_{k}^{d} & =J_{k|k-1}{\left(q\right)}^{+}\left({\dot{x}}_{k}^{d}-J_{k}\left(q\right){\dot{q}}_{k-1}^{d}\right),\\ {\ddot{q}}_{k}^{d} & =J_{k|k-1}{\left(q\right)}^{+}\left({\ddot{x}}_{k}^{d}-{\dot{J}}_{k}\left(q,\dot{q}\right)\dot{q}-J_{k}\left(q\right){\ddot{q}}_{k-1}^{d}\right), \end{array}$$ (4)

${\dot{q}}_{0}^{d}=0$ , and ${\ddot{q}}_{0}=0$ . Based on derivations of **q** ^*d*^, ${\dot{q}}^{d}$ , and ${\ddot{q}}^{d}$ , an additional optimization problem is formulated to handle full-body dynamics of the robot, contact wrench cone constraints, and torque limits as described in Kim et al. (2020), i.e.:

$$\begin{array}{cc} \underset{F_{c},{\ddot{x}}_{c},\delta \ddot{q}}{\min} & \;F_{c}^{⊤}W_{F}F_{c}+{\ddot{x}}_{c}^{⊤}W_{c}{\ddot{x}}_{c}+\delta {\ddot{q}}^{⊤}W_{\ddot{q}}\delta \ddot{q}\\ s.t.\; & \;M\left(q\right)\ddot{q}+B\left(q,\dot{q}\right)=S^{⊤}\Gamma +J_{c}^{⊤}\left(q\right)F_{c},\\ & \;{\ddot{x}}_{c}=J_{c}\left(q\right)\ddot{q}+{\dot{J}}_{c}\left(q,\dot{q}\right)\dot{q},\\ & \;\ddot{q}={\ddot{q}}^{d}+K_{p}^{h}e_{q}+K_{d}^{h}{\dot{e}}_{q}+\delta \ddot{q},\\ & \;U\left(q\right)F_{c}\geq 0,\\ & \;\Gamma_{\min}\leq \Gamma \leq \Gamma_{\max} \end{array}$$ (5)

Where $W_{F}\in S_{+}^{6m}$ , $W_{c}\in S_{+}^{6m}$ , and $W_{\ddot{q}}\in S_{+}^{n}$ are weighting matrices for the objective function. $K_{p}^{h}=\mathrm{diag}\left(k_{p_{1}}^{h},\ldots ,k_{p_{n}}^{h}\right)$ and $K_{d}^{h}=\mathrm{diag}\left(k_{d_{1}}^{h},\ldots ,k_{d_{n}}^{h}\right)$ are feedback gains in the optimization problem. $U\left(q\right)\in R^{17}$ is a matrix for expressing the contact wrench cone constraints and upper bounds of the reaction forces for smooth contact changes. **Γ**_min_ and **Γ**_max_ are the minimum and maximum torques of the actuators. Based on the optimal decision variables $F_{c}^{*}$ and $\delta {\ddot{q}}^{⋆}$ , we can provide the feedforward torque command as follows:

$${\hat{\Gamma }}^{d}=S\left(M\left(q\right){\ddot{q}}^{⋆}+B\left(q,\dot{q}\right)-J_{c}{\left(q\right)}^{⊤}F_{c}^{⋆}\right)$$ (6)

Where ${\ddot{q}}^{⋆}={\ddot{q}}^{d}+K_{p}^{h}e_{q}+K_{d}^{h}{\dot{e}}_{q}+\delta {\ddot{q}}^{⋆}$ . Using the above desired torque command ${\hat{\Gamma }}^{d}$ , we can compute the low-level joint torque command in (2).

### 2.2 Closed-Loop Analysis Without External Perturbations

We analyze the closed-loop behavior of the robotic system. Substituting (6) into (2) we get:

$$\Gamma_{\text{cmd}}=S\left(M\left(q\right)\hat{\ddot{q}}+B\left(q,\dot{q}\right)-J_{c}{\left(q\right)}^{⊤}F_{c}^{⋆}\right)$$ (7)

Where $\hat{\ddot{q}}={\ddot{q}}^{d}+{\hat{K}}_{p}e_{q}+{\hat{K}}_{d}{\dot{e}}_{q}+\delta {\ddot{q}}^{⋆}$ , ${\hat{K}}_{p}=\left(K_{p}^{h}+K_{p}\right)$ , and ${\hat{K}}_{d}=\left(K_{d}^{h}+K_{d}\right)$ . By substituting the command in (7) into the full-body dynamics in (1), the closed-loop dynamics become:

$$\left[\begin{array}{cc} M_{vv} & M_{va}\\ M_{va}^{⊤} & M_{aa} \end{array}\right]\left[\begin{array}{c} {\ddot{q}}_{v}\\ {\ddot{q}}_{a} \end{array}\right]=\left[\begin{array}{c} d_{v}\\ d_{a} \end{array}\right],$$ (8)

With

$$\begin{array}{cc} d_{v}= & S_{v}J_{c}{\left(q\right)}^{⊤}F_{c}-B_{v},\\ d_{a}= & M_{va}^{⊤}{\hat{\ddot{q}}}_{v}+M_{aa}{\hat{\ddot{q}}}_{a}+SJ_{c}{\left(q\right)}^{⊤}\left(F_{c}-F_{c}^{⋆}\right) \end{array}$$ (9)

Where subscriptions ${\left(.\right)}_{v}\in R^{6}$ and ${\left(.\right)}_{a}\in R^{n_{u}}$ denote the properties for virtual and actuated joints, respectively. Virtual joints are those corresponding to degrees of freedom for the floating base whereas actuated joints correspond to motorized robot articulations. As such, we define a selection matrix for the virtual joints, $S_{v}=\left[\begin{array}{cc} I_{6\times 6} & 0_{6\times n_{u}} \end{array}\right]\in R^{6\times n}$ . We also introduce a matrix called the Schur Complement of **M**_*aa*_ in **M**(**q**) as $M_{aa,s}=M_{vv}-M_{va}M_{aa}^{-1}M_{va}^{⊤}$ . Since **M**(**q**) is positive definite and **M**_*aa*_ is invertible, the matrix **M**_*aa*,*s*_ is also invertible. The actuated joint acceleration can be expressed as follows:

$$\begin{array}{cc} {\ddot{q}}_{a}= & M_{aa}^{-1}d_{a}+M_{aa}^{-1}M_{va}^{⊤}M_{aa,s}^{-1}\left(M_{va}M_{aa}^{-1}d_{a}-d_{v}\right)\\ = & {\hat{\ddot{q}}}_{a}+\Delta c \end{array}$$ (10)

Where $\Delta c=M_{aa}^{-1}\left(M_{va}^{⊤}\left({\hat{\ddot{q}}}_{v}-{\ddot{q}}_{v}\right)+SJ_{c}{\left(q\right)}^{⊤}\left(F_{c}-F_{c}^{⋆}\right)\right)$ . Assuming that the optimal decision variable satisfies $F_{c}=F_{c}^{⋆}$ and ${\hat{\ddot{q}}}_{v}={\ddot{q}}_{v}$ with perfect compensation for the Coriolis/centrifugal and gravitational forces, we can decouple the joint space dynamics: Δ**c** = **0**. However, the optimal decision variable $F_{c}^{⋆}$ relies on the robot model and there is no direct feedback control-loop for regulating the contact force error $F_{c}-F_{c}^{⋆}$ . Also, Δ**c** becomes significant when Centroidal dynamics are not precisely controlled due to external disturbances, since both ${\ddot{q}}_{v}$ and **F**_*c*_ significantly affect the Centroidal dynamics of the robot. So the above assumptions are frequently invalid in the real world. This is the reason to introduce the error Δ**c** in (10). Then, we can approximately decompose the closed-loop dynamics in the joint space as follows:

$${\ddot{q}}_{i}={\ddot{q}}_{i}^{f}+{\hat{k}}_{p_{i}}\left(q_{i}^{d}-q_{i}\right)+{\hat{k}}_{d_{i}}\left({\dot{q}}_{i}^{d}-{\dot{q}}_{i}\right)+\Delta c_{i}$$ (11)

Where ${\ddot{q}}_{i}^{f}={\ddot{q}}_{i}^{d}+\delta {\ddot{q}}_{i}^{⋆}$ , ${\hat{k}}_{p_{i}}=k_{p_{i}}+k_{p_{i}}^{h}$ , and ${\hat{k}}_{d_{i}}=k_{d_{i}}+k_{d_{i}}^{h}$ for all *i* ∈ {7, …, *n*}. *q*_*i*_, ${\dot{q}}_{i}$ , and ${\ddot{q}}_{i}$ represent the joint position, velocity, and acceleration values for the *i*th joint, respectively. Also, Δ*c*_*i*_ is the *i*th component of Δ**c**. The relationships between the natural frequency, *W*_*n*_, and the PD gains are defined as $W_{n}^{2}={\hat{k}}_{p_{i}}$ and $2\zeta W_{n}={\hat{k}}_{d}$ , respectively, where *ζ* denotes the damping ratio. To achieve critical damping, the derivative gain must be, ${\hat{k}}_{d_{i}}=2\sqrt{{\hat{k}}_{p_{i}}}$ . Based on this analysis, it is possible to obtain the gain matrices of the whole-body control to achieve stable closed-loop behavior.

## 3 Proposed Gain Adaptation Method

**FIGURE 2:**
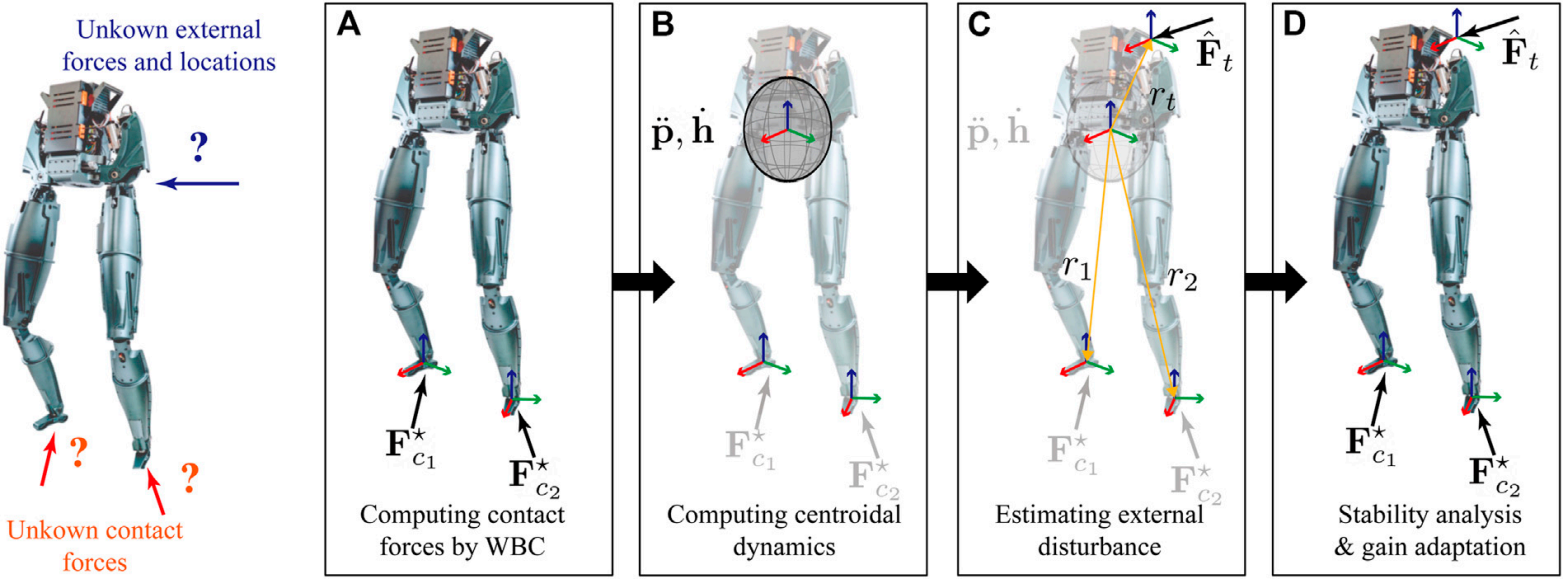
Our approach consists of: **(A)** obtaining contact forces *via* WBLC. **(B)** Computing Centroidal dynamics using the current joint configuration and velocity. **(C)** Estimating unknown external forces with respect to the robot’s body frame. **(D)** Online gain adaptation with stability analysis.

Our online gain adaption approach considers robots with unknown ground reaction forces on their feet as well as unknown external forces applied to their bodies. We use four steps to tune the feedback gains in WBC as shown in Figure 2. First, we obtain a solution for the ground reaction forces using the optimization problem (5) as shown in Figure 2A. Second, given current joint position and velocities, the centroidal dynamics are computed, discretized and approximated as shown in Figure 2B. In Figure 2C, we show the estimation of unknown external forces applied to the robot’s body using the previously computed ground contact forces and centroidal robot dynamics. Lastly, we test the stability of the robot considering the estimated forces and contacts. In turn the feedback gains are adapted to stabilize the robot under the external disturbance, as shown in Figure 2D. This section describes the detailed process of our approach.

### 3.1 Closed-Loop Behavior With External Forces

Next, we consider an additional unknown external force applied to the robot’s body, such as a force acting on the torso or pelvis. The optimization problem (5) cannot directly incorporate the unknown perturbation. Given such perturbations, the rigid-body dynamics of the robot changes as follows:

$$M\left(q\right)\ddot{q}+B\left(q,\dot{q}\right)=S^{⊤}\Gamma +J_{c}^{⊤}\left(q\right)F_{c}+J_{t}^{⊤}\left(q\right)F_{t}$$ (12)

Where $F_{t}\in R^{6}$ denotes the unknown external force acting on the robot’s body and $J_{t}\left(q\right)\in R^{6\times n}$ is the corresponding Jacobian. Based on Proposition 1, the closed-loop behavior of the system with the control torque input becomes:

$$\begin{array}{cc} {\ddot{q}}_{a}= & {\hat{\ddot{q}}}_{a}+\Delta c+M_{aa}^{-1}SJ_{t}{\left(q\right)}^{⊤}F_{t}\\ = & {\hat{\ddot{q}}}_{a}+\underset{S\psi_{t}}{\underset{︸}{\Delta c+\left(S{+E}_{m}\right)M{\left(q\right)}^{-1}S^{⊤}SJ_{t}{\left(q\right)}^{⊤}F_{t}}} \end{array}$$ (13)

Where $E_{m}=\left[\begin{array}{cc} -M_{aa,s}^{-1}M_{va}^{⊤}M_{vv}^{-1}M_{vv,s} & 0 \end{array}\right]$ . When the external disturbance is unknown, it is impossible to compute Δ**c** and **F**_*t*_, separately. Therefore, we consider the effect of the external disturbance using **S*ψ***_*t*_.

Proposition 1. *Given a matrix* $E=\left[\begin{array}{cc} E_{1} & E_{12}\\ E_{12}^{⊤} & E_{2} \end{array}\right]\in S_{++}^{n}$ *where* $E_{1}\in R^{n_{1}\times n_{1}}$ *,* $E_{2}\in R^{n_{2}\times n_{2}}$ *, and* *n* = *n*_1_ + *n*_2_ *,* $E_{2}^{-1}$ *can be expressed as* $\left(E+S_{2}\right)GS_{2}^{⊤}$ *where* **G** = **E** ^−1^ *and* $S_{2}=\left[\begin{array}{cc} 0_{n_{2}\times n_{1}} & I_{n_{2}\times n_{2}} \end{array}\right]\in R^{n_{2}\times n}$ *.*

The detailed proof of Proposition 1 is provided in Appendix. We introduce an estimated external force ${\hat{F}}_{t}={\left[{\hat{f}}_{t}^{⊤},\;{\hat{\tau }}_{t}^{⊤}\right]}^{⊤}$ satisfying the following equality: flushleft

$$S\psi_{t}=SM{\left(q\right)}^{-1}\Sigma_{a}J_{t}{\left(q\right)}^{⊤}{\hat{F}}_{t}$$ (14)

Where ${\hat{f}}_{t}\in R^{3}$ and ${\hat{\tau }}_{t}\in R^{3}$ denote the estimated force and torque of the unknown disturbance, respectively. Also, $\Sigma_{a}=S^{⊤}S=\left[\begin{array}{cc} 0_{6\times 6} & 0_{6\times n_{u}}\\ 0_{n_{u}\times 6} & I_{n_{u}\times n_{u}} \end{array}\right]$ . Unfortunately, we cannot simply express **S*ψ***_*t*_ in terms of **q** and $\dot{q}$ since we need an additional explicit mapping between ${\hat{F}}_{t}$ and these joint state variables.

Our approach is to employ robot centroidal dynamics to convert the external forces into corresponding stiffness and damping forces in the joint space. We can approximate the CoM velocity, acceleration, and the time derivative of its angular momentum with a discrete time interval Δ*t* Dai et al. (2014) as follows:

$$\begin{array}{cc} \dot{p} & \approx 2\Delta t^{-1}\left(p-p^{p}\right)-J_{\text{CoM}}\left(q^{p}\right){\dot{q}}^{p},\\ \ddot{p} & \approx \Delta t^{-1}\left(\dot{p}-J_{\text{CoM}}\left(q^{p}\right){\dot{q}}^{p}\right),\\ \dot{h}\left(q,\dot{q}\right) & \approx \Delta t^{-1}\left(h\left(q,\dot{q}\right)-h\left(q^{p},{\dot{q}}^{p}\right)\right) \end{array}$$ (15)

Where **p**, $h\left(q,\dot{q}\right)$ , **J**_CoM_(**q**) denote the position of CoM, its angular momentum, and its Jacobian, respectively. **q** ^*p*^, ${\dot{q}}^{p}$ , **p** ^*p*^, and ${\dot{p}}^{p}$ are the joint position, the joint velocity, the CoM position, and the CoM velocity in the previous time step, respectively. First, we estimate the linear force corresponding to the external disturbance, **f**_*t*_, using the centroidal dynamics and CoM acceleration:

$${\hat{f}}_{t}=M_{r}\left(\ddot{p}-g\right)-\overset{m}{\underset{\ell =1}{\sum }}f_{\ell }^{⋆}$$ (16)

Where $f_{\ell }^{⋆}\in R^{3}$ represents the linear force part for the *ℓ*-th contact among $F_{c}^{⋆}$ from WBC. Also, *M*_*r*_ and **g** denote the total mass of the robot and the gravity vector, respectively. In addition, ${\hat{\tau }}_{t}$ can be estimated by using the centroidal momentum equation and ${\hat{f}}_{t}$ .

$${\hat{\tau }}_{t}=\dot{h}\left(q,\dot{q}\right)-\overset{m}{\underset{\ell =1}{\sum }}\left(r_{\ell }\times f_{\ell }^{⋆}+\tau_{\ell }^{⋆}\right)-r_{t}\times {\hat{f}}_{t}$$ (17)

Where $\tau_{\ell }^{⋆}\in R^{3}$ denotes the torque part for the *ℓ*-th contact among $F_{c}^{⋆}$ . In turn, we can re-express the closed-loop dynamics (13) using the estimated external force ${\hat{F}}_{t}$ . We cannot decouple the closed-loop dynamics due to the term ***ψ***_*t*_.

### 3.2 The Proposed Gain Adaptation Approach

Our proposed method consists of decoupling the closed-loop dynamics by approximating ***ψ***_*t*_. Our goal is to obtain a pair of PD gains to ensure that the approximated closed-loop dynamics are critically damped, i.e.

$$\psi_{t}\approx {\tilde{K}}_{p}q+{\tilde{K}}_{d}\dot{q}+\Omega_{t}$$ (18)

Where ${\tilde{K}}_{p}=\mathrm{diag}\left({\tilde{k}}_{p_{1}},\ldots ,{\tilde{k}}_{p_{n}}\right)$ , ${\tilde{K}}_{d}=\mathrm{diag}\left({\tilde{k}}_{d_{1}},\ldots ,{\tilde{k}}_{d_{n}}\right)$ , and **Ω**_*t*_ are approximated PD gain matrices and a constant bias term, respectively. To obtain this approximation we have used the centroidal dynamics and the external force approximations specified further above. First, **p** is calculated using a first order approximation:

$$p\approx p^{p}+J_{\text{CoM}}\left(q^{p}\right)\left(q-q^{p}\right)$$ (19)

In turn the estimated external force ${\hat{f}}_{t}$ can be expressed in joint space as follows using (15) and (16):

$$\begin{array}{cc} & {\hat{f}}_{t}\left(q,\dot{q}\right)=M_{r}J_{\text{CoM}}{\left(q^{p}\right)L}_{f}\left(q,\dot{q}\right)-M_{r}g-\overset{m}{\underset{\ell =1}{\sum }}f_{\ell }^{⋆},\\ & L_{f}\left(q,\dot{q}\right)=\frac{2\left(q-q^{p}\right)}{\Delta t^{2}}-\frac{2{\dot{q}}^{p}}{\Delta t}=K_{p}^{f}q+K_{d}^{f}\dot{q}+\epsilon_{f} \end{array}$$ (20)

Where both matrices, $K_{p}^{f}=\mathrm{diag}\left(k_{p_{1}}^{f},\ldots ,k_{p_{n}}^{f}\right)$ and $K_{d}^{f}=\mathrm{diag}\left(k_{d_{1}}^{f},\ldots ,k_{d_{n}}^{f}\right)$ , represent gain matrices. Using established adaptive techniques we chose the proportional gain as $k_{p_{i}}^{f}=\eta_{i}^{f}\left(q_{i}^{d}-q_{i}^{p}\right)$ and plugging it in the second equation shown in (20), we get $k_{d_{i}}^{f}=\xi_{i}^{f}\Delta tk_{p_{i}}^{f}$ where $\eta_{i}^{f}$ and $\xi_{i}^{f}$ represent positive coefficients. We also get ***ϵ***_*f*_ as:

$$\epsilon_{f}=\left(2I-\Delta tK_{pd}^{f}\right){\ddot{q}}^{f}-K_{pd}^{f}{\dot{q}}^{p}-K_{p}^{f}q^{p}$$ (21)

Where $K_{pd}^{f}=\Delta tK_{p}^{f}+K_{d}^{f}$ . In turn Eq. 20 becomes:

$${\hat{f}}_{t}\left(q,\dot{q}\right)={\tilde{K}}_{p}^{f}q+{\tilde{K}}_{d}^{f}\dot{q}+\gamma_{f}$$ (22)

Where ${\tilde{K}}_{p}^{f}=M_{r}J_{\text{CoM}}\left(q^{p}\right)K_{p}^{f}$ , ${\tilde{K}}_{d}^{f}=M_{r}J_{\text{CoM}}\left(q^{p}\right)K_{d}^{f}$ , and $\gamma_{f}=M_{r}J_{\text{CoM}}\left(q^{p}\right)\epsilon_{f}-M_{r}g-\sum_{\ell =1}^{m}f_{\ell }^{⋆}$ .

Secondly, we estimate (15) using the first order approximation:

$$\begin{array}{cc} h\left(q,\dot{q}\right)\approx & h\left(q^{p},{\dot{q}}^{p}\right)+H_{G}\left(q^{p},{\dot{q}}^{p}\right)\left(q-q^{p}\right)+A_{G}\left(q^{p}\right)\left(\dot{q}-{\dot{q}}^{p}\right),\\ A_{G}\left(q\right)= & \frac{\partial h}{\partial \dot{q}}\left(q,\dot{q}\right),\;H_{G}\left(q,\dot{q}\right)=\overset{n}{\underset{k=1}{\sum }}\frac{\partial A_{G}^{k}\left(q\right)}{\partial q}{\dot{q}}_{k} \end{array}$$ (23)

Where $A_{G}^{k}\left(q\right)$ is the *k*th column vector of **A**_*G*_(**q**). Based on the above approximations, the estimated external torque can be expressed as follows:

$$\begin{array}{cc} {\hat{\tau }}_{t}\left(q,\dot{q}\right)= & H_{G}{\left(q^{p},{\dot{q}}^{p}\right)L}_{\tau }\left(q,\dot{q}\right)+A_{G}\left(q^{p}\right){\ddot{q}}^{f}-\overset{m}{\underset{\ell =1}{\sum }}\left(r_{\ell }\times f_{\ell }^{⋆}+\tau_{\ell }^{⋆}\right)-r_{t}\times {\hat{f}}_{t}\left(q,\dot{q}\right),\\ L_{\tau }\left(q,\dot{q}\right)= & K_{p}^{\tau }q+K_{d}^{\tau }\dot{q}+\epsilon_{\tau } \end{array}$$ (24)

Where both matrices, $K_{p}^{\tau }=\mathrm{diag}\left(k_{p_{1}}^{\tau },\ldots ,k_{p_{n}}^{\tau }\right)$ and $K_{d}^{\tau }=\mathrm{diag}\left(k_{d_{1}}^{\tau },\ldots ,k_{d_{n}}^{\tau }\right)$ , are gain matrices for estimating the external torque. More specifically, each component can be computed using adaptive control techniques in Åström and Wittenmark (2013) as: $k_{p_{i}}^{\tau }=\eta_{i}^{\tau }\left(q_{i}^{d}-q_{i}^{p}\right)$ and $k_{d_{i}}^{\tau }=\xi_{i}^{\tau }\Delta tk_{p_{i}}^{\tau }$ , where $\eta_{i}^{\tau }$ and $\xi_{i}^{\tau }$ represent positive coefficients. The term ***ϵ***_*τ*_ is described as $\epsilon_{\tau }=\left(\Delta tI-\Delta tK_{pd}^{\tau }\right){\ddot{q}}^{f}+\left(I-K_{pd}^{\tau }\right){\dot{q}}^{p}-K_{p}^{\tau }q^{p}$ where $K_{pd}^{\tau }=\Delta tK_{p}^{\tau }+K_{d}^{\tau }$ . Suppose **r**_*ℓ*_ and **r**_*t*_ are constant then we can re-write the estimated ${\hat{\tau }}_{t}$ as:

$${\hat{\tau }}_{t}\left(q,\dot{q}\right)={\tilde{K}}_{p}^{\tau }q+{\tilde{K}}_{d}^{\tau }\dot{q}+\gamma_{\tau }$$ (25)

Where ${\tilde{K}}_{p}^{\tau }=H_{G}\left(q^{p},{\dot{q}}^{p}\right)K_{p}^{\tau }-skew\left(r_{t}\right){\tilde{K}}_{p}^{f}$ and ${\tilde{K}}_{d}^{\tau }=H_{G}\left(q^{p},{\dot{q}}^{p}\right)K_{d}^{\tau }-skew\left(r_{t}\right){\tilde{K}}_{d}^{f}$ . The detailed ***γ***_*τ*_ is

$$\gamma_{\tau }=H_{G}\left(q^{p},{\dot{q}}^{p}\right)\epsilon_{\tau }-r_{t}\times \gamma_{f}-\overset{m}{\underset{\ell =1}{\sum }}\left(r_{\ell }\times f_{\ell }^{⋆}+\tau_{\ell }^{⋆}\right)+A_{G}\left(q^{p}\right){\ddot{q}}^{f}.$$ (26)

We can construct ${\hat{F}}_{t}$ using selection matrices $S_{f}={\left[I_{3\times 3},\;0_{3\times 3}\right]}^{⊤}$ and $S_{\tau }={\left[0_{3\times 3},\;I_{3\times 3}\right]}^{⊤}$ as follows:

$${\hat{F}}_{t}=S_{f}{\hat{f}}_{t}\left(q,\dot{q}\right)+S_{\tau }{\hat{\tau }}_{t}\left(q,\dot{q}\right)={\tilde{K}}_{p}^{s}q+{\tilde{K}}_{d}^{s}\dot{q}+\gamma^{s}$$ (27)

Where ${\tilde{K}}_{p}^{s}=S_{f}{\tilde{K}}_{p}^{f}+S_{\tau }{\tilde{K}}_{p}^{\tau }$ , ${\tilde{K}}_{d}^{s}=S_{f}{\tilde{K}}_{d}^{f}+S_{\tau }{\tilde{K}}_{d}^{\tau }$ , and ***γ*** ^*s*^ = **S**_*f*_ ***γ***_*f*_ + **S**_*τ*_ ***γ***_*τ*_. Using the above estimated external force ${\hat{F}}_{t}$ , we approximate ***ψ***_*t*_ as follows:

$$\begin{array}{cc} \psi_{t}\approx & M{\left(q^{p}\right)}^{-1}\Sigma_{a}J_{t}\left(q^{p}\right)\left({\tilde{K}}_{p}^{s}q+{\tilde{K}}_{d}^{s}\dot{q}+\gamma^{s}\right)\\ \approx & {\hat{Z}}_{p}q+{\hat{Z}}_{d}\dot{q}+{\hat{\Omega }}_{t} \end{array}$$ (28)

Where ${\hat{Z}}_{p}$ and ${\hat{Z}}_{d}$ are diagonal matrices. We define ${\hat{Z}}_{p}=\mathrm{diag}\left(z_{p_{1}},\ldots ,z_{p_{n}}\right)$ and ${\hat{Z}}_{d}=\mathrm{diag}\left(z_{d_{1}},\ldots ,z_{d_{n}}\right)$ where $z_{p_{i}}$ and $z_{d_{i}}$ are the *i*th diagonal components of $M{\left(q^{p}\right)}^{-1}\Sigma_{a}J_{t}\left(q^{p}\right){\tilde{K}}_{p}^{s}$ and $M{\left(q^{p}\right)}^{-1}\Sigma_{a}J_{t}\left(q^{p}\right){\tilde{K}}_{d}^{s}$ , respectively. The last term takes the form ${\hat{\Omega }}_{t}=M{\left(q^{s}\right)}^{-1}J_{t}{\left(q^{p}\right)}^{⊤}\gamma^{s}$ . Since ${\hat{K}}_{p}$ , ${\hat{K}}_{d}$ , ${\hat{Z}}_{p}$ , and ${\hat{Z}}_{d}$ are diagonal matrices, the closed-loop dynamics in actuated joint level space becomes:

$${\ddot{e}}_{a}=\left({\hat{K}}_{p}^{a}-{\hat{Z}}_{p}^{a}\right)e_{a}+\left({\hat{K}}_{d}^{a}-{\hat{Z}}_{d}^{a}\right){\dot{e}}_{a}+{\bar{\Omega }}_{t,a}$$ (29)

Where $e_{q_{a}}=q_{a}^{d}-q_{a}$ , ${\dot{e}}_{a}={\dot{q}}_{a}^{d}-{\dot{q}}_{a}$ , and ${\ddot{e}}_{a}=S\left({\ddot{q}}^{d}+\delta {\ddot{q}}^{⋆}\right)-{\ddot{q}}_{a}$ . Also, we define ${\hat{K}}_{p}^{a}=S{\hat{K}}_{p}=\mathrm{diag}\left({\hat{k}}_{p_{7}},\ldots ,{\hat{k}}_{p_{n}}\right)$ , ${\hat{K}}_{d}^{a}=S{\hat{K}}_{d}=\mathrm{diag}\left({\hat{k}}_{d_{7}},\ldots ,{\hat{k}}_{d_{n}}\right)$ , ${\hat{Z}}_{p}^{a}=\mathrm{diag}\left(z_{p_{7}},\ldots ,z_{p_{n}}\right)$ , and ${\hat{Z}}_{d}^{a}=\mathrm{diag}\left(z_{d_{7}},\ldots ,z_{d_{n}}\right)$ , and ${\bar{\Omega }}_{t,a}=S\left({\hat{\Omega }}_{t}-{\hat{Z}}_{p}q^{d}-{\hat{Z}}_{d}{\dot{q}}^{d}\right)$ .

We define the state $\nu ={\left[e_{a}^{⊤},\;{\dot{e}}_{a}^{⊤}\right]}^{⊤}$ and express the state-space model of the above dynamics equation as follows:

$$\dot{\nu }=\underset{V}{\underset{︸}{\left[\begin{array}{cc} 0_{n} & I_{n}\\ -{\hat{K}}_{p}^{a}+{\hat{Z}}_{p}^{a} & -{\hat{K}}_{d}^{a}+{\hat{Z}}_{d}^{a} \end{array}\right]}}\nu +\left[\begin{array}{c} 0_{n\times 1}\\ {\bar{\Omega }}_{t,a} \end{array}\right].$$ (30)

Properly choosing $\eta_{i}^{f}$ , $\xi_{i}^{f}$ , $\eta_{i}^{\tau }$ , and $\xi_{i}^{\tau }$ , we can make ${\bar{\Omega }}_{t,a}$ negligible. Then, the matrix **V** should be Hurwitz to guarantee that the closed-loop state model is exponentially stable. To compute the characteristic equation of the matrix **V**, we express the determinant of the matrix **V** − *λ* **I**_2*n*_ as follows:

$$\begin{array}{cc} \det\left(\left[\begin{array}{cc} -\lambda I_{n} & I_{n}\\ -{\hat{K}}_{p}^{a}+{\hat{Z}}_{p}^{a} & -{\hat{K}}_{d}^{a}+{\hat{Z}}_{d}^{a}-\lambda I_{n} \end{array}\right]\right) & =\lambda^{-1}\det\left({\hat{K}}_{d}-{\hat{Z}}_{\dot{q}}+\lambda^{-1}\left({\hat{K}}_{p}-{\hat{Z}}_{q}\right)+\lambda I_{n\times n}\right)\\ & =\det\lambda^{-1}\underset{X}{\underset{︸}{\left(\left({\hat{K}}_{d}^{a}-{\hat{Z}}_{d}^{a}\right)+\lambda^{-1}\left({\hat{K}}_{p}^{a}-{\hat{Z}}_{p}^{a}\right)\right)}}+I_{n}, \end{array}$$

Which means that   det(**V** − *λ* **I**_2_) =  det(*λ* ^−1^ **X** + **I**_*n*_). It is complicated to obtain analytic forms of the eigenvalues of **V**. For this reason, we can compute the determinant above using the Leibniz formula described in Bhatia (2013).

$$\det\left(\lambda^{-1}X+I_{n}\right)=1+\lambda^{-1}trace\left(X\right)+O\left({\left(\lambda^{-1}\right)}^{2}\right)$$ (31)

Where O(.) represents the big-O notation. Using the above formulation, the characteristic equation can be re-written as:

$$\det\left(V-\lambda I_{2n}\right)=1+\lambda^{-1}trace\left(X\right)+O\left({\left(\lambda^{-1}\right)}^{2}\right)=0.$$ (32)

All solutions of the above characteristic equation *λ* should be negative to guarantee that the matrix **V** is Hurwitz. Such analysis could be conducted *via* numerical computation given the gain matrices. However, in this paper, we aim at adaptive feedback gains of the controller so that we obtain ${\tilde{K}}_{p}^{a}={\hat{Z}}_{p}^{a}$ and ${\tilde{K}}_{d}^{a}={\hat{Z}}_{d}^{a}$ . In turn the term $O\left({\left(\lambda^{-1}\right)}^{2}\right)$ vanishes. We can obtain the analytical expression of the approximated characteristic equation as follows:

$$\lambda^{2}+\lambda trace\left({\hat{K}}_{p}^{a}-{\tilde{K}}_{p}^{a}\right)+trace\left({\hat{K}}_{d}^{a}-{\tilde{K}}_{d}^{a}\right)=0.$$ (33)

This condition will allow us to make the closed-loop system stable.

Condition 1. *The following condition makes the matrix* **V** *Hurwitz:* $\sum_{i=7}^{n}{\hat{k}}_{p_{i}}-z_{p_{i}}>0$ *and* $\sum_{i=7}^{n}{\hat{k}}_{d_{i}}-z_{d_{i}}>0$ *. Without any adaptation, these condition should be fulfilled to make the closed-loop system asymptotically stable given desired feedback gains.*

We can approximately decouple the dynamics of (29) in the joint space as follows:

$${\ddot{q}}_{i}+\left({\hat{k}}_{d_{i}}-z_{d_{i}}\right){\dot{q}}_{i}+\left({\hat{k}}_{p_{i}}-z_{p_{i}}\right)q_{i}={\tilde{F}}_{i}^{⋆}$$ (34)

Where ${\tilde{F}}_{i}^{⋆}={\ddot{q}}_{i}^{f}+{\hat{k}}_{p_{i}}q_{i}^{d}+{\hat{k}}_{d_{i}}{\dot{q}}_{i}+{\hat{\Omega }}_{t}\left(i\right)$ . We enforce that the joint space dynamics are critically damped given $z_{p_{i}}$ and $z_{d_{i}}$ for all *i* ∈ {7, …, *n*}. For the above system, the following relationships are obtained in terms of the natural frequency and the damping ratio: ${\hat{k}}_{p_{i}}-z_{p_{i}}={\bar{W}}_{n}^{2}$ and ${\hat{k}}_{d_{i}}-z_{d_{i}}=2\zeta {\bar{W}}_{n}$ . Since we already know all gains and coefficients, ${\hat{k}}_{p_{i}}$ , ${\hat{k}}_{d_{i}}$ , $z_{p_{i}}$ , and $z_{d_{i}}$ , *ζ* can be determined a priori. We can compute adaptive variables $\varepsilon_{p_{i}}$ and $\varepsilon_{d_{i}}$ to make ${\bar{W}}_{n}=W_{n}^{d}$ and *ζ* = *ζ* ^*d*^.

$$\begin{array}{cc} \varepsilon_{p_{i}} & ={\left(W_{n}^{d}\right)}^{2}+z_{q_{i}}-{\hat{k}}_{p_{i}},\\ \varepsilon_{d_{i}} & =2\zeta^{d}\sqrt{{\hat{k}}_{p_{i}}+\varepsilon_{p_{i}}-z_{q_{i}}}+z_{d_{i}}-{\hat{k}}_{d_{i}}. \end{array}$$ (35)

Condition 2. *Since* *W*_*n*_ *is a positive real number, it is always true that* ${\hat{k}}_{p_{i}}+\varepsilon_{p_{i}}-z_{p_{i}}>0$ *and* ${\hat{k}}_{d_{i}}+\varepsilon_{d_{i}}-z_{d_{i}}>0$ *in the adaptive system. In such case:* $\sum_{i=7}^{n}{\hat{k}}_{p_{i}}+\varepsilon_{p_{i}}-z_{p_{i}}>0$ *and* $\sum_{i=7}^{n}{\hat{k}}_{d_{i}}+\varepsilon_{d_{i}}-z_{d_{i}}>0$ *. However, if there are no* $\varepsilon_{p_{i}}$ *and* $\varepsilon_{d_{i}}$ *satisfying the equation above, the motion bandwidth cannot be stably achievable under the external force.*

Based on the previous adaptation scheme, we replace the WBLC $k_{p_{i}}^{h}$ , $k_{d_{i}}^{h}$ by ${\bar{k}}_{p_{i}}^{h}$ , ${\bar{k}}_{d_{i}}^{h}$ in the optimization problem where ${\bar{k}}_{p_{i}}^{h}=k_{p_{i}}^{h}+\varepsilon_{p_{i}}$ and ${\bar{k}}_{d_{i}}^{h}=k_{d_{i}}^{h}+\varepsilon_{d_{i}}$ . These feedback gains are limited in practice. Since relying on the contact forces, the adaptive gains might abruptly increase during contact transition or applying an impact force. For such reason, we consider the following additional condition:

Condition 3. *We set the ranges of* $\varepsilon_{p}\in \left[\varepsilon_{p}^{\min},\;\varepsilon_{p}^{\max}\right]$ *and* $\varepsilon_{d}\in \left[\varepsilon_{d}^{\min},\;\varepsilon_{d}^{\max}\right]$ *for real implementation in robotic systems.*

After testing all conditions, we update the gains in WBLC. Then, the control command torque is obtained without further modifications given the task specifications. Algorithm 1 summarizes the entire process of how to update the feedback gains in WBLC.

Algorithm 1 Proposed Gain Adaptation Algorithm

**Data:** **q**, $\dot{q}$ , **q** ^*p*^, ${\dot{q}}^{p}$ , $x_{k}^{d}$ , ${\dot{x}}_{k}^{d}$ , ${\ddot{x}}_{k}^{d}\;\forall k\in \left\{1,\ldots ,\;N\right\}$

**Result:** **Γ**_cmd_

${\hat{F}}_{c}^{⋆}\leftarrow$ WBLC in (5)
**J**_CoM_ ← with **q** ^*p*^

${\tilde{K}}_{p}^{f}$ , ${\tilde{K}}_{d}^{f}$ , ***γ***_*f*_ ← (22)
**h**, **H**_*G*_, **A**_*G*_ ← with **q** ^*p*^ and ${\dot{q}}^{p}$

${\tilde{K}}_{p}^{\tau }$ , ${\tilde{K}}_{d}^{\tau }$ , ***γ***_*τ*_ ← (25)

${\tilde{K}}_{p}^{s}$ , ${\tilde{K}}_{d}^{s}$ , ***γ***_*s*_ ← (27)

${\hat{Z}}_{p}$ , ${\hat{Z}}_{d}\;\leftarrow$ (28)

$\varepsilon_{p_{i}}$ , $\varepsilon_{d_{i}}\;\forall i\in \left\{7,\ldots ,n\right\}\;\leftarrow$ (35)
**if** *Condition 1, 2, and 3 are satisfied* **then**

${\bar{k}}_{p_{i}}^{h}=k_{p_{i}}^{h}+\varepsilon_{p_{i}}$ , ${\bar{k}}_{d_{i}}^{h}=k_{d_{i}}^{h}+\varepsilon_{d_{i}}\;\forall i\in \left\{7,\ldots ,n\right\}$

**end**

$K_{p}^{h}$ and $K_{d}^{h}$ in (5) $\leftarrow \;{\bar{k}}_{p_{i}}^{h}$ and ${\bar{k}}_{d_{i}}^{h}$

**Γ**_cmd_ ← with the updated $K_{p}^{h}$ and $K_{d}^{h}$

## 4 Simulations and Experiments

**FIGURE 3:**
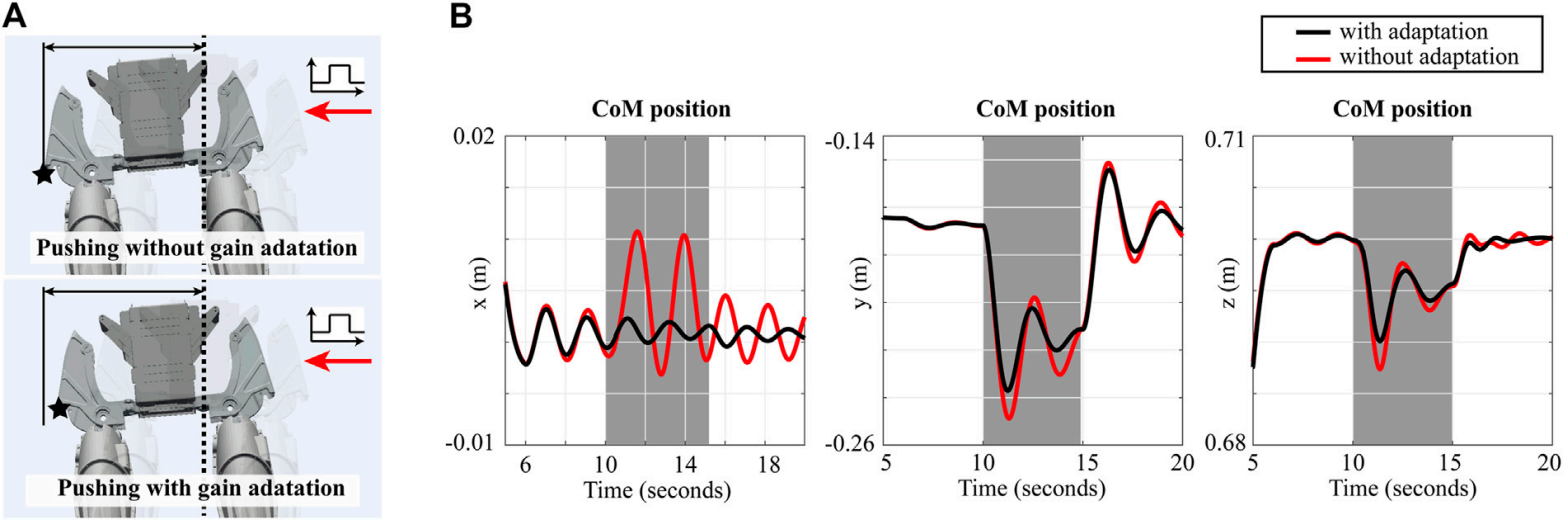
**(A)** Snapshots of control simulations: the upper and lower figures show balancing behaviors with and without gain adaptation, respectively. A black star marks the edge point on the hip shell as a measurement reference. **(B)** Comparison between the positions of the robot’s CoM using standard WBLC versus adaptive WBLC, where the desired x and y positions of the CoM is the center position between two feet [3.80, ×, 10^–5^, −0.167] m, and the desired height of the CoM is 0.7 m.

In this section, our proposed adaptive approach is validated by demonstrating both simulations and experiments using a 10 DOF liquid-cooled bipedal robot called *Draco2* and a full-body humanoid robot called *Valkyrie*. Since *Draco2* is a line-feet biped robot, it is hard for it to maintain its balance during the double support phase robustly. So, in section 4.1, we focus on showing how to stabilize the robot’s body during the double support phase while an external force is applied to its pelvis, as shown in Figure 3. In turn we demonstrate experimentally that our online gain adaptation method can be applied in real-time to a physical platform as discussed in section 4.2. Using *Valkyrie* which uses flat feet for balancing, we implement walking simulations to verify the effectiveness of the proposed online gain adaptation approach when there exists long-term perturbations instead of fast impacts. This study is described in section 4.3. We employ our open-source software architecture called PnC^1^ (Planning and Control).

### 4.1 *Draco2* Simulations

Using *Draco2*, we implement three simulations to show that the proposed approach makes the robot more stable: *1*) pushing the pelvis in the double support phase, *2*) pushing the pelvis while swinging CoM position, *3*) applying an impact force to the pelvis in the double support. For whole-body control, we define these tasks: **x**_CoM_, **x**_base,__o_, **x**_feet_, and **x**_joint_, which represent tasks for controlling the position of the robot’s CoM, the orientation of its floating base, the position and orientation of its feet, and the joint configuration, respectively.

#### 4.1.1 Pushing Force Applied to the Pelvis

The first simulation aims to control the position of the robot’s CoM while applying a pushing force at the pelvis. We define the desired location of the CoM as [3.80, ×, 10^–5^, −0.167, 0.7] m in the double support phase. The external force is applied to the y-direction of the pelvis with an amplitude of 12 N during the interval [10, 15] s. We design the appropriate bounds for the gain margins as $\varepsilon_{p}^{\max}=350$ , $\varepsilon_{p}^{\min}=-100$ , $\varepsilon_{d}^{\max}=60$ , and $\varepsilon_{d}^{\min}=-10$ .

**FIGURE 4:**
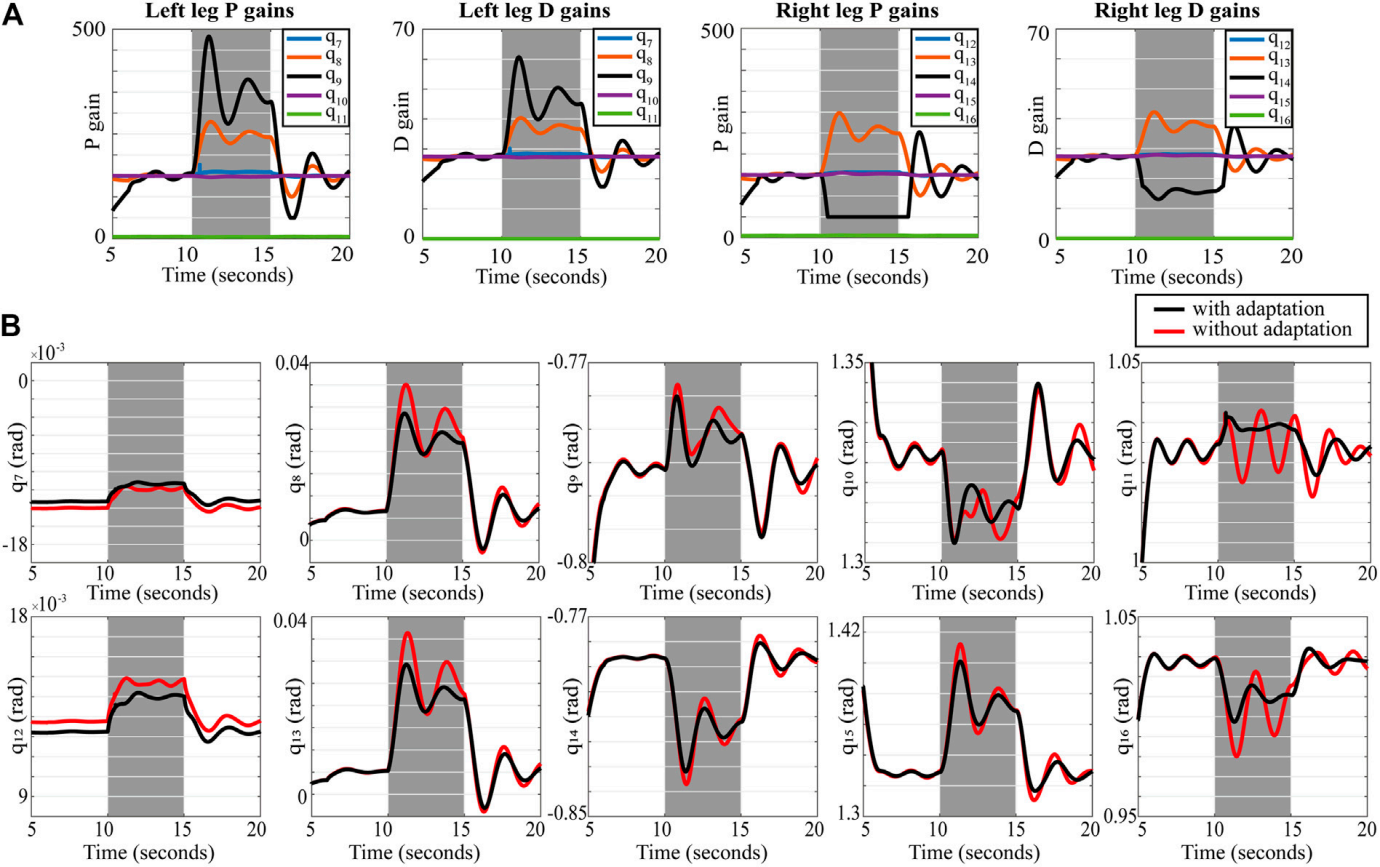
Simulation results of pushing the pelvis: **(A)** The adapted gains of the WBLC in the joint level. **(B)** The configuration of the robot controlled by WBLC.

As the snapshots show in Figure 3A, gain adaptation reduces the adverse effects of external forces on the robot’s behavior. Figure 3B shows the CoM positions when the robot is controlled with and without the proposed gain adaptation scheme. For a more precise analysis, we compare the maximum variations of the robot’s CoM position: max(**x**_CoM_) − min(**x**_CoM_). Without the proposed approach, [0.013 9, 0.081 0, 0.012 4] m is the maximum amplitude of the variation. When using our proposed adaptation approach the amplitude reduces to [0.029, 0.069 5, 0.009 8] m. In addition, we compare the maximum distances of the position of the CoM $d_{\max}^{\text{CoM}}=\|x_{\text{CoM}}^{d}-x_{\text{CoM}}^{\text{far}}\|$ where $x_{\text{CoM}}^{\text{far}}$ denotes the farthest position from the desired position while the external force is acting on the robot. The values of $d_{\max}^{\text{CoM}}$ with and without the proposed adaptive approach are 0.070 9 m and 0.083 5 m, respectively. Therefore $d_{\max}^{\text{CoM}}$ with the proposed approach is 84.91% of that without our method. Although the difference is small, it is significant for legged robots having a small support polygon such as *Draco2*. The proposed approach results in the adaptation of the feedback gains in WBLC as shown in Figure 4A. Also, the configuration of the robot for each case is represented in Figure 4B. As shown in the results, the adapted gains contribute to stabilize the robot so that the configuration becomes less oscillatory. More specifically, the settling times of the simulations with and without the proposed approach are 18.16 and 10.2 s, respectively.

#### 4.1.2 Pushing Force While Swinging

**FIGURE 5:**
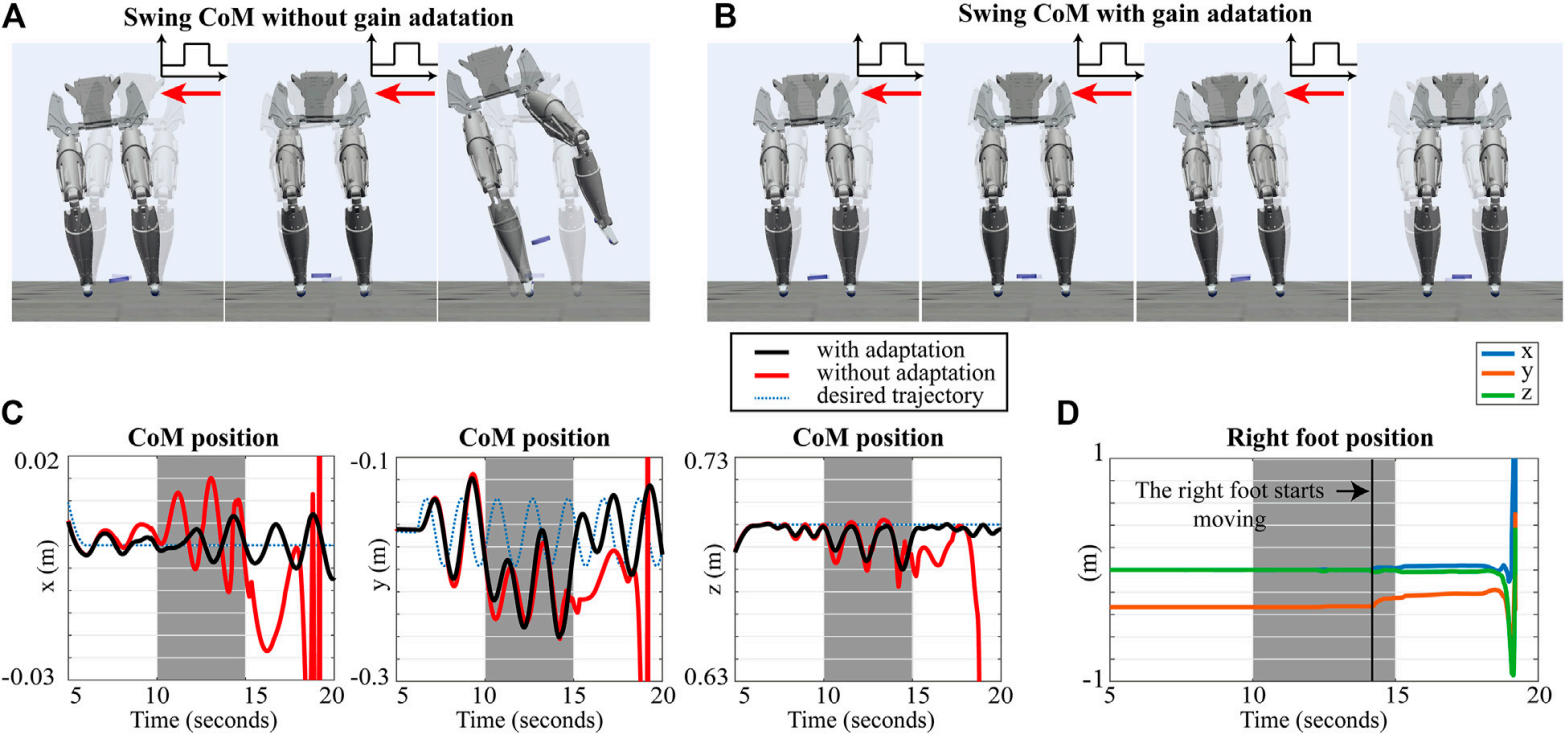
Simulation results of CoM swing with unknown external force: **(A)** Snapshots of the simulation swinging CoM without the proposed gain adaption. **(B)** Snapshots of the simulation with the proposed gain adaptation. **(C)** The Positions of the robot’s CoM while performing a swinging motion: x, y, and z position of the CoM, **(D)** The right foot position without the adaptive gains.

In this second simulation, we implement a behavior consisting of swinging the position of the robot’s CoM in double support phase while an external force is acting on the pelvis. We generate a sinusoidal reference for the CoM with 0.03 m amplitude and 0.5 Hz frequency. Figures 5A,B show snapshots of the simulations. As shown in Figure 5A, the controller with fixed gains falls due to the effect of the external force. On the other hand, the adaptive gains prevent the robot from losing balance, as shown in Figure 5B. The positions of the CoM controlled by WBLC with and without adaptive gains are compared in Figure 5C. The robot with the adaptive gains can track the trajectory of the CoM while maintaining solid contacts with the ground. However, employing WBLC without adaptation fails to control the position of the CoM as shown in Figure 5C, and as a result the right foot starts moving away from contact around 14 s as shown in Figure 5D.

#### 4.1.3 Impact Force

**FIGURE 6:**
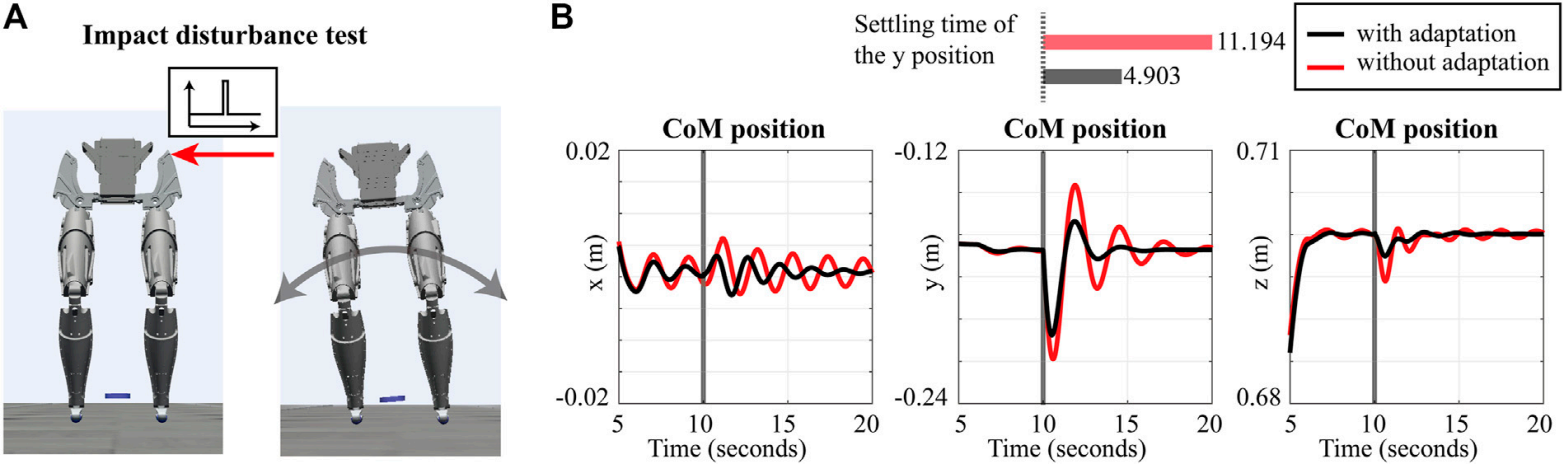
Simulation results of impact test: **(A)** the desired position of the CoM is the same as that for the pushing scenario. An impact force is applied to the robot with an amplitude of 700 N and duration 0.01 at 10 s. **(B)** The position of the robot’s CoM.

The last simulation compares the response of the robot due to an impact force with and without the proposed adaptive control approach. An impact force with an amplitude 700 N over 0.01 s from 10 s is applied. Figure 6 shows the position of the CoM in response to the impact force, and the impact timing is showing as marked lines. We compare the settling times of the y position of the CoM, which are 11.194 and 4.903 s without and with the proposed approach, respectively. Also, the peak of the y position of the CoM with adaptive gains, − 0.207 6 m, is smaller than that without the proposed approach, − 0.219 0 m. Based on these results, it is verified that the proposed approach makes the robot more robust to impact forces.

#### 4.1.4 Addition Simulations

Here, we increase the amplitude of the external disturbances with respect to the previous simulations to analyze the effectiveness of our method. Firstly, we increase the external pushing force from −12 N to −18 N with respect to the forces used in Section 4.1.1. Without the proposed adaptation, the robot falls when applying −14 N due to the effect of the pushing force. However, the robot with the adapted gain scheme keeps its balance while tolerating external forces up to −17 N. The robot falls when applying −18 N. Secondly, we increase the pushing force from −10 N to −17 N while swinging the CoM position as shown in the simulation in Section 4.1.2. The robot with fixed gains loses its balance when the pushing force is −12 N. By contrast, our method enables the robot to swing the CoM position robustly while increasing the external force up to −16 N. These results indicate that the robot’s balance is more fragile when applying the external force during dynamic motions rather than static ones. Lastly, we repeat the impact test of Section 4.1.3 with different amplitudes of the impact forces. The robot without our method slips and falls when the amplitude of the impact is −1500 N. In contrast, our gain adaptation scheme keeps the robot standing up to −1900 N of impact force. Overall, these results confirm that our proposed gain adaptation method yields more robust balancing capabilities against external disturbances than using fixed gains.

For more detailed analysis of the results, we calculate the Integral Absolute Error (IAE) of the CoM task with respect to the external forces during the perturbation period:

$$IAE=\int_{t_{i}}^{t_{f}}\|x_{\text{com}}^{d}\left(t\right)-x_{\text{com}}\left(t\right)\|dt$$ (36)

**FIGURE 7:**
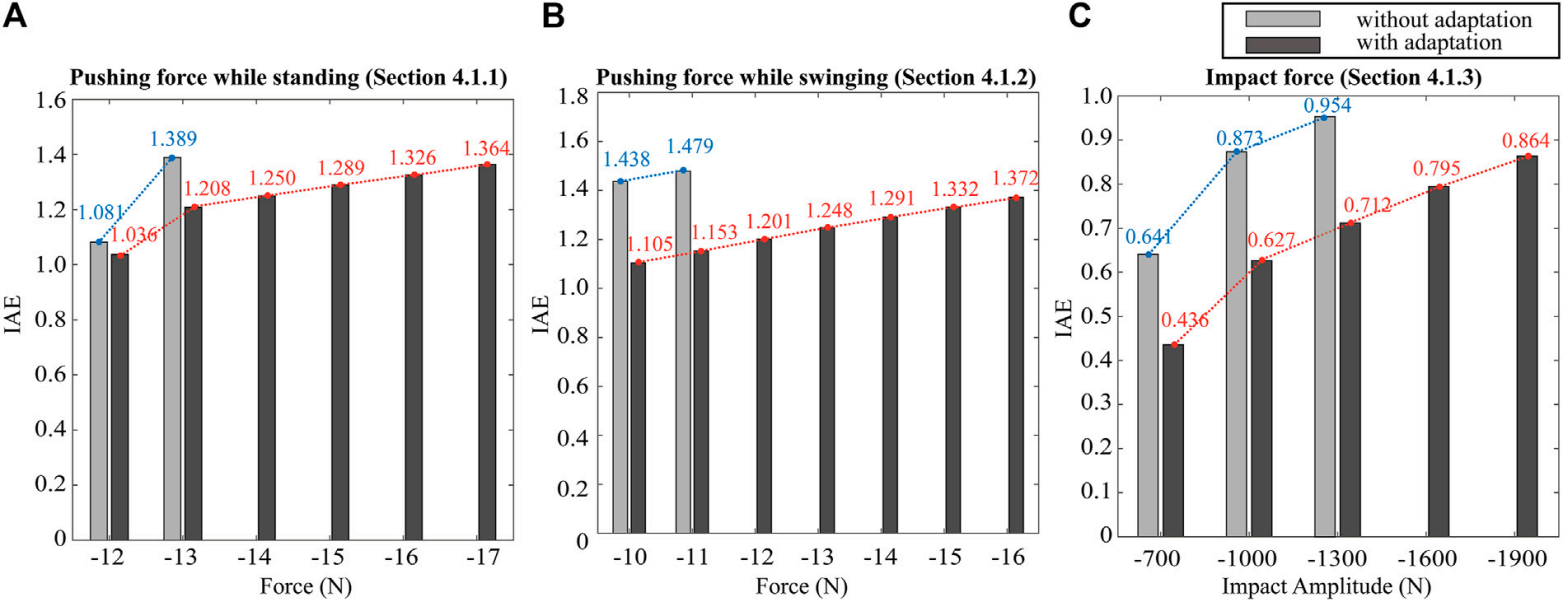
IAE values corresponding to simulation results with different levels of external disturbances: The simulation scenarios are those described in Sections 4.1.1 **(A)**, 4.1.2 **(B)**, and 4.1.3 **(C)**. To calculate the IAE values of the impact simulation **(C)**, we use the same time interval [10, 15] to cases **(A**,**B)**.

Where [*t*_*i*_, *t*_*f*_] denotes the time interval for the perturbation. Figure 7 shows the IAE values for all simulation results concerning the various amplitudes of the external disturbances. We repeat the same simulation scenarios shown in Sections 4.1.1 (push while standing: Figure 7A), 4.1.2 (push while swinging the CoM position: Figure 7B), and 4.1.3 (impact while standing: Figure 7C). As shown in Figure 7, our proposed method results in smaller IAE values compared to fixed-gain WBLC. The comparison of the IAE values shows that our proposed approach reduces the task tracking error and makes the robot more robust regarding disturbance rejection.

**FIGURE 8:**
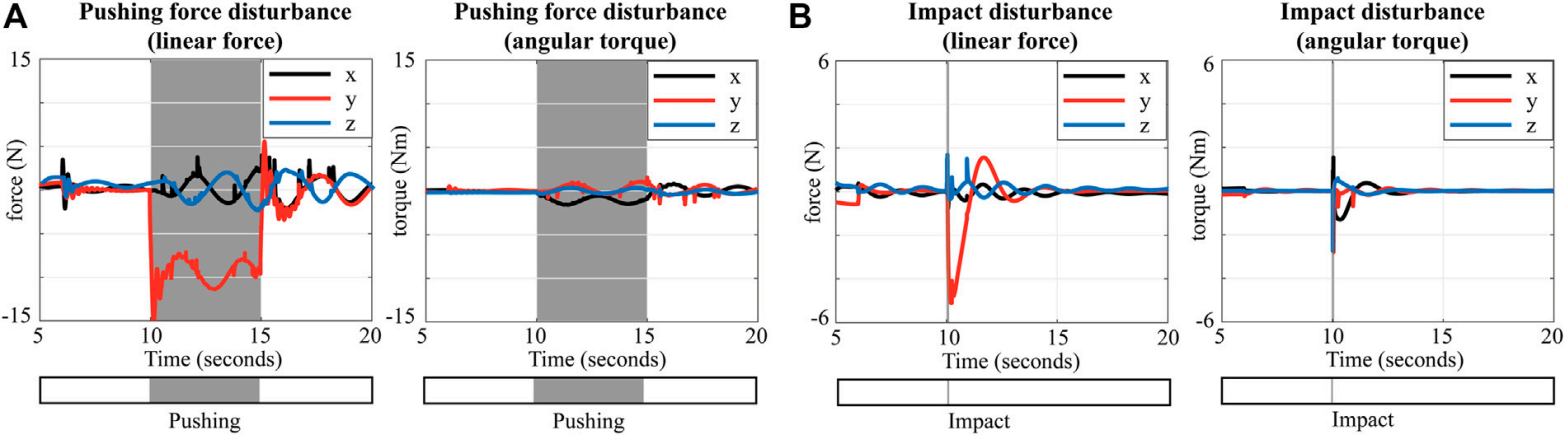
Estimation of unknown external forces using the contact force formula provided by WBLC: **(A)** Pushing force disturbance (− 12 N in the y direction), **(B)** Impact force disturbance (− 700 N in the y direction).

Figures 8A,B represent the estimated disturbances ${\hat{F}}_{t}$ when applying the pushing force (Section 4.1.1) and the impact disturbance (Section 4.1.3), respectively. The mean of the estimated force ${\hat{F}}_{t}$ in Figure 8A is ${\hat{F}}_{t,mean}=\left[-1.966,\;-8.626,\;0.667,\;-2.981,\;0.483,\;0.297\right]$ N. The estimated amplitude is approximately 72% of the real external force. Therefore our estimate of the pushing force disturbance is a rough approximate, but it has the advantage that it does not require external F/T sensors to measure the contact forces. By contrast, a limitation of this estimation is that the impact disturbance cannot be precisely estimated as shown in Figure 8B, since we employ the centroidal dynamics and the WBLC formulas to estimate ${\hat{F}}_{t}$ . The peak value of the estimated impact in the lateral direction ${\hat{F}}_{t,\left[y\right]}$ is only −5.2 N. Additional impact models and information such as impact time are required to compute the accurate impact between rigid-bodies Stewart (2000), however, this is out of the scope in this paper.

### 4.2 *Draco2* Experiments

**FIGURE 9:**
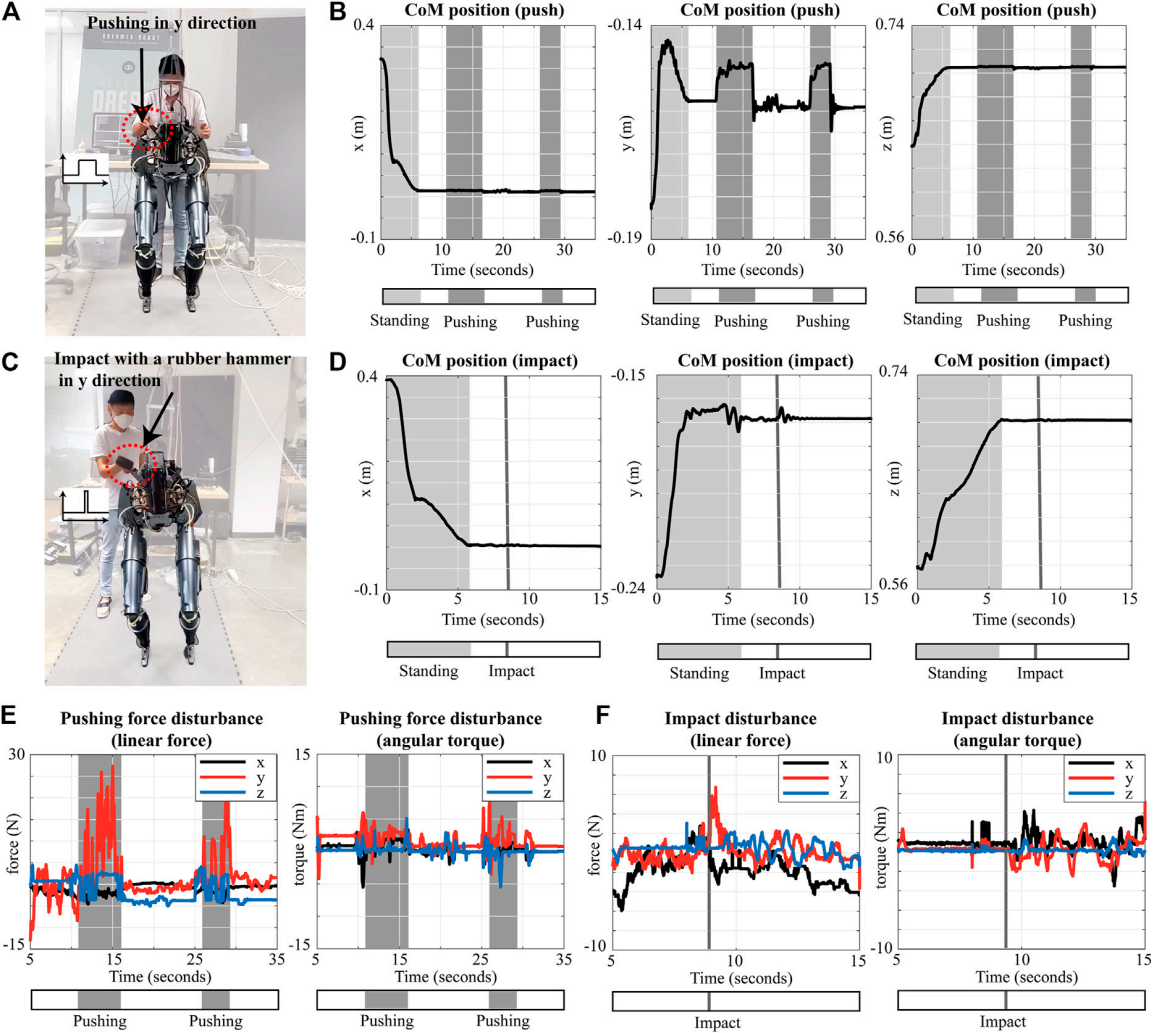
Experiment results with the same scenarios with the simulations in 4.1.1 and 4.1.3: pushing **(A**,**B)** and impacting **(C**,**D)** the pelvis of *Draco2* in double support phase. The estimated external disturbance ${\hat{F}}_{t}$ is computed when pushing **(E)** and impacting **(F)** the robot.

In this section, we verify the proposed approach using the real *Draco2* robot. The main goal of these experimental evaluations is to show that the simulation results can be reproducible in real hardware. We select two experimental scenarios, which are to keep the balance under pushing and impact forces and evaluate them in the real hardware as shown in Figure 9. The robot is standing upright in the double support phase, and we push and hit the handle at the pelvis of the robot.

The positions of the CoM are shown in Figure 9 for both experimental scenarios. As shown in Figure 9, the robot is standing up while tracking the predefined desired CoM position, which is identical to the desired CoM position described in Sections 4.1.1, 4.1.3. For the first scenario, we push the robot at 10.6 and 25.8 s twice, which is shown as marked zones in Figure 9B. Although the robot’s CoM position has fluctuated in the y-direction, the robot does not fall and keeps its balance. Figure 9D shows the position of the CoM for the second scenario. We hit the robot’s pelvis with the rubber hammer at 8.5 s, which is shown as marked lines in Figure 9D. Note that the proposed approach makes the robot stable and keeps its balance in double support against the external perturbations as anticipated. Without our adaptation approach, it is not possible to keep the robot balanced when disturbed as shown in our Supplementary Video. Since we could not directly measure the pushing and impact disturbances, we estimate ${\hat{F}}_{t}$ as shown in Figures 9E,F. More specifically, the peak and mean values of the estimated pushing external disturbances in two pushing time intervals shown in Figure 9E are ${\hat{F}}_{t,\left[y\right]}^{\text{peak}}=\left[27.14,\;22.41\right]$ N and ${\hat{F}}_{t,\left[y\right]}^{\text{mean}}=\left[10.8,\;8.6\right]$ N, respectively. Figure 9F shows the estimation of the external disturbance with a peak in the y direction of 6.8 N. The experimental results show that our approach is effective in the real hardware under similar conditions to the simulations.

### 4.3 *Valkyrie* Simulations

For simulated walking behaviors, we generate a reference trajectory for the robot’s CoM using the three-dimensional Divergent Component of Motion (DCM) Englsberger et al. (2013). In addition, we define multiple tasks to control the robot *Valkyrie* using WBLC: **x**_CoM_, **x**_AM_, **x**_pelvis,__o_, **x**_rf_, and **x**_lf_ which represent the tasks for controlling the position of the CoM, the body’s angular momentum, the orientation of the pelvis, and the position and orientation of the right and left feet, respectively. Additionally, we assume that the desired angular momentum change is zero during the planning and control calculations.

#### 4.3.1 DCM Planner and Contact Transition

This section briefly introduces a locomotion planner based on DCM and the parameters that we use. DCM is defined as a point that lies ahead of the position of the CoM:

$$\xi =x_{\text{CoM}}+b{\dot{x}}_{\text{CoM}}$$ (37)

Where *b* > 0 denotes the time constant of the DCM dynamics. We generate a desired DCM trajectory as discussed in Englsberger et al. (2013) and convert it to a CoM trajectory. Given walking parameters, the reference DCM can be computed at a time step *t*, ***ξ***_ref_(*t*), in turn we can obtain ${\dot{x}}_{\text{CoM}}^{d}\left(t\right)$ and $x_{\text{CoM}}^{d}\left(t\right)$ using Euler integration:

$$\begin{array}{cc} {\dot{x}}_{\text{CoM}}^{d}\left(t\right)= & -\frac{1}{b}\left(x_{\text{CoM}}^{d}\left(t-\Delta t\right)-\xi_{\text{ref}}\left(t\right)\right),\\ x_{\text{CoM}}^{d}\left(t\right)= & x_{\text{CoM}}^{d}\left(t-\Delta t\right)+{\dot{x}}_{\text{CoM}}^{d}\left(t\right)\Delta t \end{array}$$ (38)

Where Δ*t* is the time increment. The reference DCM and feet trajectories are interpolated using a polynomial function and B-spline.

One of the advantages of WBLC is that we do not need to change the optimization problem to achieve smooth contact transitions. Using WBLC, it is possible to ensure smooth transitions between the support phases by changing the weighting matrices in the objective function in the optimization problem. The weighting matrix for the contacts **W**_*c*_ are interpolated as follows:

$$W_{c}^{\text{update}}=\left(1-s\left(t\right)\right)W_{c}^{\text{old}}+s\left(t\right)W_{c}^{\text{new}}$$ (39)

Where $W_{c}^{\text{old}}$ and $W_{c}^{\text{new}}$ denote the weighting matrices before and after contact transition. s:R↦[0,1] can be a monotonously increasing function of time from 0 to 1.

#### 4.3.2 Pushing Force While Walking

We implement simulations of our method when walking with external pushing forces. Here *Valkyrie* walks five steps forward. An unknown lateral force is applied to the pelvis of the robot with an amplitude of 100 N during the interval [6.5, 8.5] s. For DCM planning, the nominal height of the CoM is 1.015 m. The single support swing time and the transition time are set to 0.75 and 0.45 s, respectively. The length of the subsequent steps is set to 0.2 m.

**FIGURE 10:**
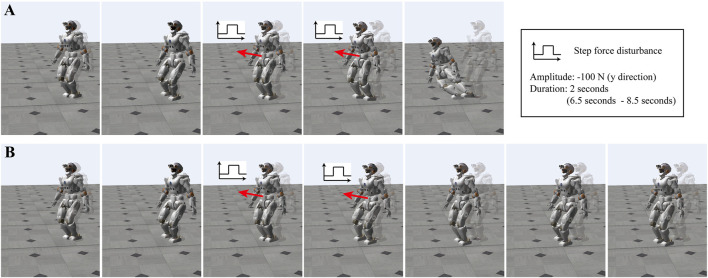
Snapshots of simulations for walking while an external force exists: **(A)** walking without the proposed gain adaptation, **(B)** walking with the online gain adaptation.

**FIGURE 11:**
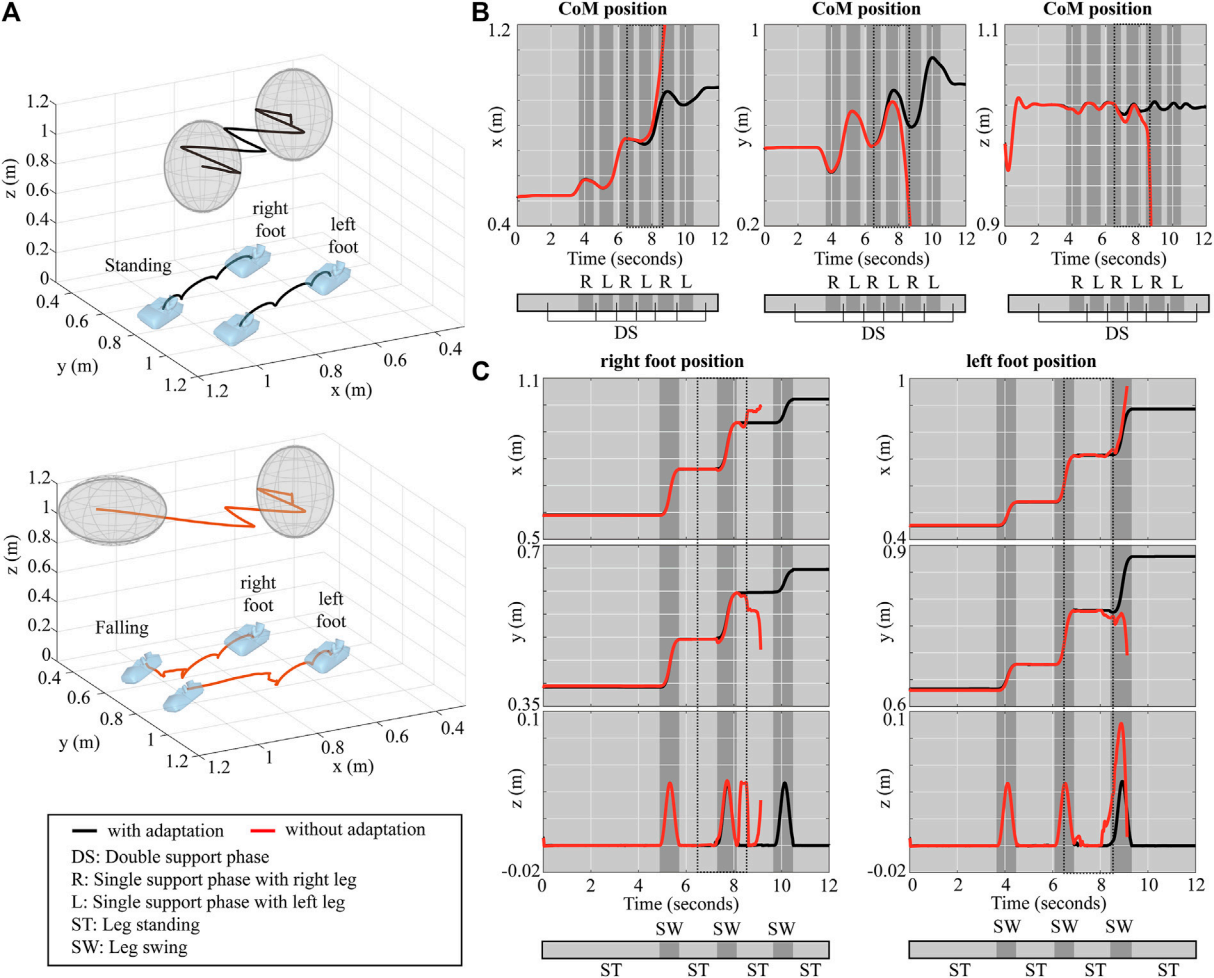
Simulation results of walking with an external force: **(A)** positions of CoM and feet in Cartesian coordinate, **(B)** the robot’s CoM position, **(C)** the robot’s feet positions.

Figure 10 shows snapshots of walking simulations. Without our gain adaptation approach, the robot tilts and falls due to the disturbances, as shown in Figure 10A. In contrast, the robot is able to walk forward against the external forces when using our proposed method (*see* Figure 10B). The detailed simulation results are shown in Figure 11. In Figure 11A, we depict the positions of the CoM and both feet in Cartesian space. Figure 11A shows that the robot with gain adaptation can track the preplanned trajectories before and after the perturbation. The exact CoM position and foot locations are shown in Figures 11B,C. After standing upright, the initial CoM position of the robot is [0.521 2, 0.512 2, 1.020 7] m. With the adapted gains, the robot’s CoM reaches the goal destination [0.950 7, 0.763 2, 1.017 8] m. The center position of the feet also changes from [0.520 7, 0.512 1, 0] m to [0.954 2, 0.763 3, 0] m. However, without the proposed approach, the robot’s feet start to move slightly in the second swing phase of the right foot, as shown in Figure 11C. Also, the CoM position cannot be controlled from that moment. Then the right foot detaches from the ground at 8.2 s although it should remain on the floor. Note that these simulations show robust performance of the proposed underlying approach against unknown external force disturbances.

## 5 Conclusion

This paper presents extensive analysis, algorithms, and experimentation for the robust and adaptive employment of whole-body controllers in legged robots. The proposed approach devises a strategy to adapt feedback control gains of WBLC in response to disturbances. When a pushing force or impact occurs, the desired feedback gains are computed given nominal natural frequency and damping ratio. Subsequently, we test the theory to achieve stability both in simulation and experimental evaluations. Thus, our proposed approach is validated in both simulation and experimentation during double support balancing under pushing and impacting forces. Also, we implement walking simulations using a DCM planner to validate our method. The simulation and experimental results show that the adaptive WBLC enables legged robots to keep their balance against unknown external disturbances. We summarize the results of Draco simulations to show the improved performance in Table 1. Although we previously addressed dynamic walking under pushing forces using WBLC, that work was not adaptive and in turn relied solely on replanning. In contrast, in this new work we have developed a framework for adaptive WBLC that may require less reliance on replanning and provide better stability and robustness. In the future, we will extend this work to loco-manipulation behaviors and under multiple external force disturbances.

**TABLE 1:** Summary of Draco simulations.

	Max. disturbance			Integral Absolute Error (IAE)			Settling time
	Push (stand)	Push (swing)	Impact (stand)	Push: −13 N (stand)	Push: −11 N (swing)	Impact: −1300 N (stand)	Impact: −1300 N (stand)
wo/	−13 N	−11 N	−1300 N	1.389	1.479	0.954	9.61 s
w/	−17 N	−16 N	−1900 N	1.208	1.153	0.712	5.90 s
ratio	130.7%	145.5%	146.2%	86.9%	77.9%	74.6%	61.3%

## Data Availability

The original contributions presented in the study are included in the article/Supplementary Material, further inquiries can be directed to the corresponding authors.

## Footnotes

1 PnC package: https://github.com/junhyeokahn/PnC

## Appendix

### Proof of Proposition 1

Since **E** is positive definite, **E**_1_, **E**_2_, **E**_1,*s*_, and **E**_2,*s*_ are invertible where **E**_1,*s*_ and **E**_2,*s*_ are Schur Complements of **E**_1_ and **E**_2_, respectively. It is possible to express the partitioned matrices of **G** as follows:

$$G=\left[\begin{array}{cc} G_{1} & G_{12}\\ G_{12}^{⊤} & G_{2} \end{array}\right]=\left[\begin{array}{cc} E_{2,s}^{-1} & -E_{1}^{-1}E_{12}E_{1,s}^{-1}\\ -E_{1,s}^{-1}E_{12}^{⊤}E_{1}^{-1} & E_{1,s}^{-1} \end{array}\right]$$
(40)

Then, we get the following formula by comparing the expressions of **E** with each Schur Complements:

$$\begin{array}{cc} E_{2}^{-1} & =E_{1,s}^{-1}-E_{2}^{-1}E_{12}^{⊤}E_{2,s}^{-1}E_{12}E_{2}^{-1}\\ & =G_{2}-E_{1,s}^{-1}E_{12}^{⊤}E_{1}^{-1}E_{2,s}G_{12}\\ & =\left(E+S_{2}\right)GS_{2}^{⊤} \end{array}$$
(41)

Where $E=\left[\begin{array}{cc} -E_{1,s}^{-1}E_{12}^{⊤}E_{1}^{-1}E_{2,s} & 0 \end{array}\right]\in R^{n_{2}\times n}$ .
